# Li-Fraumeni Syndrome With Six Primary Tumors—Case Report

**DOI:** 10.1155/2024/6699698

**Published:** 2024-05-10

**Authors:** Dejan Stojiljković, Ana Cvetković, Andrej Jokić, Dijana Mirčić, Sanja Mihajlović, Ana Krivokuća, Marija Đorđić Crnogorac, Lazar Glisic

**Affiliations:** ^1^Department of Surgery, Surgical Oncology Clinic, Institute for Oncology and Radiology of Serbia, Belgrade, Serbia; ^2^Faculty of Medicine, University of Belgrade, Belgrade, Serbia; ^3^Department of Anesthesiology With Reanimatology and Intensive Care Unit, Institute for Oncology and Radiology of Serbia, Belgrade, Serbia; ^4^Department for Experimental Research and Genetics, Institute for Oncology and Radiology of Serbia, Belgrade, Serbia; ^5^Department of Obstetrics and Gynecology, University Clinic Ulm, Faculty of Medicine, University of Ulm, Ulm, Germany

**Keywords:** case report, Li-Fraumeni, mitotane, p53

## Abstract

Li-Fraumeni syndrome (LFS) is a cancer predisposition syndrome associated with a high, lifetime risk of a broad spectrum of cancers caused by pathogenic germline TP53 mutations. Numerous different germline TP53 mutations have been associated with LFS, which has an exceptionally diverse clinical spectrum in terms of tumor type and age of onset. Our patient has developed six asynchronous tumors to date: a phyllode tumor of the breast, a pheochromocytoma, a rosette-forming glioneuronal tumor (RGNT), an adrenocortical carcinoma (ACC), a ductal carcinoma of the breast, and a thymoma. The occurrence of such a number of rare tumors is sporadic even among in the population of patients living with cancer predisposition syndromes. In this instance, the omission of pretest genetic counseling and thorough family tree analysis prior to selecting the test led to the oversight of an underlying TP53 likely pathogenic mutation (classified as Class 4). This emphasizes the necessity for such counseling to prevent overlooking crucial genetic information. Neglecting this step could have had profound implications on the patient's treatment, particularly considering the early onset and occurrence of multiple tumors, which typically raise suspicion of a hereditary component. The implications for family members must be considered.

## 1. Introduction

Multiple primary neoplasms (MPNs) are defined as two or more primary neoplasms in which each tumor is not an extension, recurrence, or metastasis of the other. They are rare in the general population and are sporadically described in case reports. Our review of the literature found only a few patients with three or more neoplasms [1–3]. In recent years, as the life expectancy of cancer patients has increased due to better diagnostic and therapeutic methods, the incidence of MPNs has been increased. MPNs are most common in syndromes associated with genetic disorders. Li-Fraumeni syndrome (LFS) is extremely rare; it is estimated that it affects 1 in 5000 to 1 in 20,000 families [3, 4]. The incidence of suspected TP53 mutation ranges from 1 in 3555 to 1 in 5476 individuals, but the number is unknown due to a lack of genetic screening [5]. Here, we present our case of a patient with LFS who developed six primary tumors by the age of 33 years.

## 2. Case Description

In September 2005, at the age of 17, the patient started oncology treatment when she was hospitalized for a large left breast tumor and underwent tumorectomy. Phyllode glandulae mammae was diagnosed in the postoperative pathohistologic report. Two months later, the patient presented for a left-sided simplex mastectomy with a sentinel lymph node biopsy (pathohistology report: lymph nodes 0+/3). Two years later, in October 2007, a secondary reconstruction of the left breast was performed. In the same year, the patient was hospitalized for palpitations and headaches. The diagnosis of pheochromocytoma was suspected after a thorough laboratory examination and was subsequently confirmed by a postoperative pathohistological report.

In 2007, *BRCA1* testing was performed in the commercial genetic laboratory. It was ordered because of her early-onset breast cancer diagnosis. The whole coding region of BRCA1 gene was analyzed, and the report showed benign and likely benign genetic variants in *BRCA1* gene obtained by Sanger sequencing, the only method available in 2007 in Serbia. The report identified benign and likely benign genetic variants within the BRCA1 gene. However, due to the absence of genetic counselling teams in Serbia and limited access to comprehensive genetic testing centers, further examination for other hereditary syndromes was not pursued.

After an uneventful pregnancy in 2009, she experienced neurological deficits manifested as severe headaches, nausea, and ataxia, which progressively worsened, leading the patient to seek medical attention in 2014. The patient's magnetic resonance imaging (MRI) scan showed a cystic lesion in the region of the vermis, cerebellum, and roof of the fourth ventricle. In December 2014, the patient underwent craniotomy with the extirpation of the tumor mass. The postoperative pathohistological report confirmed a rosette-forming glioneuronal tumor (RGNT). Preoperative whole-body computed tomography (CT) revealed a nodular lesion in the left adrenal gland. Genetic testing was negative for hereditary pheochromocytoma, but there was a mutation in the ARMC5 gene responsible for bilateral macronodular hyperplasia of the adrenal glands. Subsequent fluorodeoxyglucose-positron emission tomography showed an accumulation of radiopharmaceuticals in the lesion, but the patient declined surgery because of a planned pregnancy.

After an uncomplicated pregnancy in 2016/2017, the patient was referred to her physician in the postpartum period because of a palpable mobile mass corresponding to the previously described adrenal lesion, now measuring 130 × 110 × 170 mm. Left-sided adrenalectomy was performed with adequate preoperative substitution therapy, and the pathohistology report confirmed an adrenocortical carcinoma (ACC). Chemotherapy was started with EDP protocol (etoposide, doxorubicin, and cisplatin) and mitotane, followed by lifelong replacement therapy with hydrocortisone. Unfortunately, a few days after the start of chemotherapy, the patient developed signs of an Addisonian crisis.

Laboratory results suggested mitotane toxicity with folate and free thyroxine deficiency, elevated gamma-glutamyl transferase, and hyperlipidemia. The endocrinology consultation resulted in additional therapy with fludrocortisone, after which the patient's condition improved. Therapy evaluation was performed after Cycles III and VI, which showed no dissemination or recurrence of the disease. Treatment was continued with mitotane. Two years later, the treatment was discontinued according to the protocol [6] as the patient was free of disease recurrence.

In December 2018, during a regular check-up, the CT showed a suspicious lesion in the thymus, described as residual tissue. Due to the growth of the thymic lesion and the appearance of three nodular lesions in the left lung parenchyma, a thymectomy was performed in May of 2020. Extemporaneous analysis was positive for malignant tissue. It was decided to perform the cisplatin-based hyperthermic intrathoracic chemotherapy (HITHOC) procedure. The pathohistological report confirmed a type B2 thymoma. The sample of nodular lung lesion was confirmed to be normal lung parenchyma.

In subsequent periods, regular check-ups showed no progression in the size of the remaining nodular lung lesions until May 2021, when the CT scan showed a change in size. Video-assisted thoracoscopic surgery was performed to remove the lesions in the upper left lobe of the lung. The pathohistology report confirmed the metastasis of ACC. Mitotane treatment was restarted. The goal was to achieve plasma levels in the therapeutic range of 14–20 mg/L.

**Figure 1:**
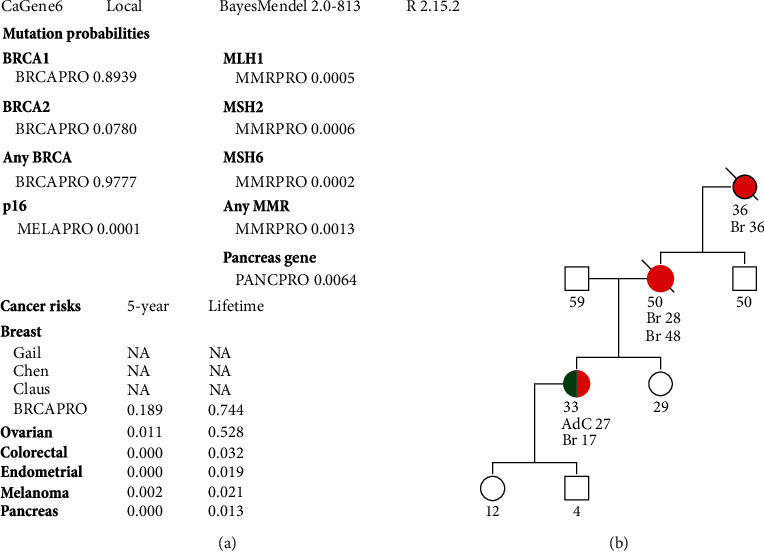
(a) Assessment of gene mutation probability and (b) family tree analysis. Source: Đorđić-Crnogorac, Marija. “Family tree analysis.” 12 Nov. 2022, accessed 27 Jan. 2023. Source: Đorđić-Crnogorac, Marija. “Assessment of the patient's probability of having a BRCA gene mutation, BRCAPRO.” 12 Nov. 2022, accessed 27 Jan. 2023.

At this point, the patient was referred by the Department for Medical Oncology and the Department for Surgical Oncology to the Department for Genetic Counselling for Hereditary Cancers at the Institute for Oncology and Radiology of Serbia that was established in 2017. The patient was received by the genetic counselling team, and previous genetic testing results were reassessed. The patient was declared to be *BRCA*1 negative based on the genetic report from 2007. Nevertheless, the carrier probability (CP) was recalculated in BRCAPRO (Figure 1(a)) which showed almost 100% CP for *BRCA* genes. Since *BRCA1* was negative, *BRCA2* testing was indicated. However, besides CP calculation and high burden of breast cancers in the family (Figure 1(b); the mother was diagnosed with bilateral breast cancer (age 28 and age 48) and the grandmother was diagnosed with the breast cancer at the age of 36), the high probability of LFS was established during the pretest genetic counselling session because of the complex personal medical history. The NGS gene panel that we offered included *BRCA1*, *BRCA2*, *ATM*, *CDH1*, *CHEK2*, *PALB2*, *PTEN*, *TP53*, and *STK11* genes. The patient signed informed consent for testing with a detailed explanation of the procedure and the course of the analysis (approved by the ethics committee of the institute) and agreed on the panel testing. After signing the informed consent, the patient was directed to blood sampling. The deoxyribonucleic acid (DNA) sample for the germline genetic analysis was isolated from the patient's whole blood lymphocytes according to the usual protocol.

Next-generation sequencing was used to analyze mutation status of the genes on the Illumina MiSeq Sequencing System (Illumina). The Nextera DNA library preparation kit was used in combination with TruSight® cancer panel (Illumina, San Diego, USA) to detect germline alterations in coding regions of genes and regions containing exon and intron boundaries.

Analysis and classification of detected gene variants were done using the software Geneticist Assistant (Soft Genetics) and Illumina Variant Interpreter (Illumina). The result showed a likely pathogenic mutation (Class 4) NM_000546.6(TP53):c.376-2A>G in a heterozygote state. The detected mutation is an intron variant that impacts the splice site, and its consequence leads to faulty or absent protein synthesis. The presence of the mutation was visually confirmed using Integrative Genomic Viewer (IGV). The variant allele frequency showed low probability of genetic mosaicism. Its classification was performed according to the American College of Medical Genetics and Genomics (ACMG) recommendation for variant classification. Other genes were negative for pathogenic/likely pathogenic genetic variants.

After germline mutation in *TP53* was detected, we referred the patient back to the surgical and medical oncology departments of the institute.

In November 2021, the patient was admitted for mammography and breast ultrasound because of palpable lesions in the right breast, followed by a biopsy, which confirmed an invasive ductal carcinoma (IDC). In the following month, the patient underwent a right subcutaneous radical mastectomy with primary breast reconstruction with an endoprosthesis. The pathohistological report was carcinoma ductale invasivum glandulae mammae. Three months later, in February 2022, a routine reexamination revealed a recurrence of the disease, this time in the right lung. Given the previous diagnosis of ACC metastasis, the surgeon did not perform a preoperative biopsy. The patient underwent a right lower lobectomy and atypical resection of the middle lobe of the lung. The pathology report confirmed that it was again a metastasis of ACC (see Table 1 for the patient's timeline).

At the last follow-up, the patient was free of local recurrence and distant metastases while on mitotane therapy.

## 3. Discussion

### 3.1. Strengths and Limitations

The strength of our case report lies in its individuality due to the high number of primary tumors and the course of treatment and its complications. The limitations, as with all case reports, are the lack of generalizability, the lack of a representative population sample, and the lack of concrete conclusions.

### 3.2. Discussion

LFS is a rare autosomal dominant hereditary disorder that predisposes carriers to cancer development. It is associated with germline mutations in the p53 tumor suppressor gene, which encodes a transcription factor (p53) that generally regulates the cell cycle and prevents genomic mutations.

**Figure 2:**
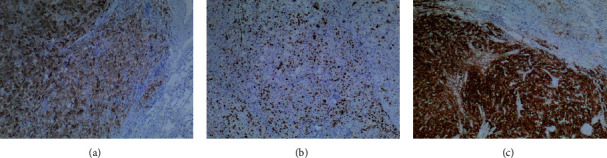
Immunohistochemistry staining of adrenocortical metastasis: (a) inhibin+, (b) Ki67%, and (c) synaptophysin+. Photo: Medić S. Belgrade, 2023.

LFS is diagnosed in patients who meet all three classic clinical criteria or have a heterozygous germline pathogenic variant in TP53. The following are the classic clinical criteria: a sarcoma diagnosed before the age of 45 years, a first-degree relative with any cancer diagnosed before the age of 45 years and a first- or second-degree relative with any cancer diagnosed before the age of 45 years, or a sarcoma diagnosed at any age (Figure 2).

Our patient has developed six histologically distinct tumors, which is extremely rare even in the population with this disorder. Hisada et al. found four primary tumors in approximately 2% of Li-Fraumeni population [7].

Phyllode tumor is a sporadic breast tumor, with an incidence between 0.3% and 0.9% of all breast tumors [8], but much higher in patients diagnosed with LFS [9]. They are classified into three grades: benign, borderline, or malignant. All phyllode tumors, whether they are cancerous or not, grow quickly. Surgical treatment to remove the tumor with a free margin is acceptable, with no difference in recurrence from a wider margin. However, younger age and malignant phyllode tumor are the two highest risk factors for recurrence.

**Figure 3:**
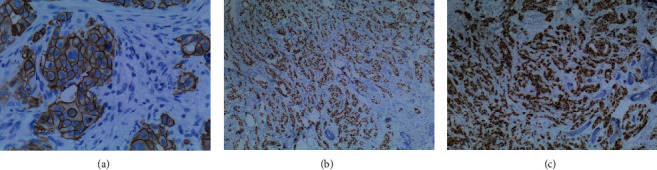
Immunohistochemistry staining of breast ductal carcinoma: (a) HER2+, (b) ER+, and (c) PR+. Photo: Medić S. Belgrade, 2023.

Carriers of TP53 mutations (Class 4 or 5) have an increased risk of developing osteosarcoma, soft tissue sarcomas, brain tumors, ACC, and leukemia. The presence of likely deleterious mutations in the TP53 gene in women increases the risk of breast cancer by up to 85% by the time patients reach 60 years of age [10, 11]. It is estimated that 3%–8% of women diagnosed with breast cancer under the age of 30 years carry a germline TP53 mutation [12]. The most common type of breast cancer is IDC [13], about 75% of all breast cancers, which is also true for patients with LFS. Local surgical treatment in patients with LFS should follow the same guidelines as in the general population, based on the clinical and pathologic features of the breast cancer and other medical comorbidities. However, in the absence of major contraindications, mastectomy may be preferred to breast-conserving surgery to avoid the need for adjuvant radiation, as radiation may predispose patients with LFS to secondary malignancies [5] (Figure 3).

ACC originating in the cortex of the adrenal gland is a rare malignancy with an incidence of 0.7–2.0 cases/million population/year [14]. Approximately 3%–10% of LFS-associated cancers are ACCs, suggesting that germline TP53 mutations confer a significant relative risk increase [15]. The course of treatment in this case was primarily influenced by the patient's refusal of treatment. Studies suggest the role of estrogens by observing a probable relative increase in the diagnosis of ACC during pregnancy, and in vitro studies confirm the growth-promoting effects of estrogen on the ACC cell line NCI-H295 [16–18]. In this case, 2.5 years resulted in significant progression of the disease. At the time of surgery, the patient was in Stage II of the disease, which has a 5-year survival rate of 58%–64% [19], but after it was discovered that the lesions in the lung parenchyma were in fact metastases of ACC. The patient was restaged to Stage IV disease with a 5-year survival rate of 0%–17% [20]. After the initial diagnosis, the patient started chemotherapy with EDP protocol and mitotane, which resulted in an Addisonian crisis. As a derivative of the insecticide dichlorodiphenyltrichloroethane, mitotane exhibits adrenolytic and cytotoxic activity. It induces the cytochrome P450 3A4 (CYP3A4), which leads to lower blood levels of many drugs and is associated with significant toxicity, including dizziness, vertigo, central nervous system disturbances, and gastrointestinal symptoms, which may reduce patient compliance to therapy.

After 2 years, mitotane was discontinued [6]. The first metastases of ACC occurred 1 year after treatment. Mitotane was restarted after surgery. The progression of the disease was confirmed by diagnostic imaging, which led to appropriate surgical procedures. At her last check-up, the patient was free of disease recurrence while still on mitotane therapy.

Pheochromocytomas are neuroendocrine tumors derived from adrenal chromaffin cells or similar cells in the extra-adrenal sympathetic or parasympathetic paraganglia, respectively. The prevalence of pheochromocytoma and paraganglioma may range from 1:6500 to 1:2500 cases per year [21, 22]. Although ACC is common in Li-Fraumeni patients, the same cannot be said for pheochromocytoma. They are rare, even in atypical presentations of the syndrome [23, 24]. Genetic testing has ruled out a hereditary component. Diagnosis is based on signs and symptoms of catecholamine excess, including hypertension, palpitations, headache, sweating, and pallor. It is confirmed by biochemical evidence of catecholamine production by the tumor. CT or MRI is recommended for initial tumor localization, with MRI preferred in patient radiation-sensitive due to concerns about radiation exposure. Surgery is the primary treatment, and laparoscopic surgery is now the first choice for resection of adrenal and extra-adrenal tumors (Figure 2).

Thymomas are tumors that arise from the thymic epithelial cells, which are involved in directing maturation of T cells. The overall incidence is 0.13 per 100,000 persons/year [25]. Their occurrence in LFS is rare, with one study presenting a single case of thymoma among 286 p53 mutation carriers [26]. In most cases, they tend to invade locally and spread along the surrounding serous membranes. There are no specific therapies for LFS-associated thymoma, and management is consistent with general guidelines for management. Surgery is the mainstay of treatment with a 10-year survival rates of 80%, 78%, 75%, and 42% for Stages I, II, III, and IV, respectively, with an R0 resection [27]. Radiotherapy has its place in selected cases, mainly in patients with Stage III or R1-2 residual [26] disease, but not in patients who are sensitive to radiation, as was the case here. Platinum-based chemotherapy remains the standard of care for patients with advanced disease [26]. Therefore, we decided to perform a combination of surgery and HITHOC. This procedure results in less toxic effects than systemic chemotherapy while providing adequate local disease control in patients with Stage IVa thymoma or pleural recurrence of thymoma [28].

In 2002, first described RGNT as a rare and novel type of glioneuronal tumors [24]. RGNT usually arises from the wall of the fourth ventricle as slow growing midline lesions. They typically present with slowly progressive cerebellar deficits, headache, and ataxia over several months to years, although patients rarely present with acute symptoms. In Li-Fraumeni patients, brain tumors are a common presentation with an incidence of approximately 10%–15% [24]. The distribution of diagnoses is relatively skewed toward specific brain tumor entities, including astrocytoma, supratentorial primitive neuroectodermal tumor (PNET), and choroid plexus carcinoma (CPC). Our patient developed headaches that persisted for several weeks, followed by ataxia, which prompted her to consult her physician. The diagnosis was made by MRI and confirmed by a pathohistological report. Surgery remains the treatment of choice, with gross total resection being the primary recommendation and subtotal resection as an alternative. The prognosis is favorable, although recurrence may occur.

This case showed how the lack of pretest genetic counseling and comprehensive family tree analysis before test selection resulted in overlooking a potentially significant TP53 likely pathogenic mutation (classified as Class 4). This underscores the crucial role of genetic counseling in avoiding such oversights and ensuring all pertinent genetic information is accounted for. Neglecting to address these considerations could profoundly impact the patient's treatment, particularly considering the early onset and frequent occurrence of tumors, often suggestive of a hereditary influence. Overlooking information on germline mutations could result in missed treatment opportunities with potentially devastating consequences. Moreover, the implications for family members must not be overlooked. Ensuring the health of family members is crucial, as proactive genetic testing and preventive measures can be initiated alongside routine medical check-ups.

### 3.3. Rationale for Conclusion

#### 3.3.1. “Take-Away” Lesson

It is important to suspect a hereditary disorder in patients who develop early or multiple tumors because early detection of genetic syndromes could influence decisions about diagnostic and therapeutic modalities, significantly improving patient survival and quality of life.

#### 3.3.2. Patient Perspective

I was a relatively young patient when I started treatment, so I had no idea what to expect. I thought I was in remission after my first breast surgery. I consider myself a positive person, but there is no peace in my life as more and more tumors have appeared in my life. Even if they told me that I was completely cured, I doubt that I would be able to keep my mind off the regular check-ups, follow-ups, medications, and hospitals.

#### 3.3.3. Informed Consent

The patient was informed properly about the purpose of the case report and gave written consent.

## Figures and Tables

**Table 1:** Patient's timeline.

September 2005. Admitted for hospital treatment due to a large left breast tumor and underwent a tumorectomy.
October 2005. Admitted for a left-sided simplex mastectomy with a sentinel lymph node biopsy because the pathohistology report confirmed it was malignant tumor.
October 2007. Admitted for a secondary reconstruction of the left breast.
December 2007. Admitted due to palpitations and headaches, tested positive for pheochromocytoma, and underwent a right-sided adrenalectomy.
In 2009. Pregnancy carried out.
December 2014. Admitted to a hospital due to progressive ataxia and severe headaches. The MR confirmed a brain tumor, and the patient underwent craniotomy with the extirpation of the tumor mass.
In 2016/2017. Pregnancy. In the postpartum period, a palpable mobile mass was discovered and subsequently confirmed to be adrenocortical carcinoma.
In 2017. The patient gave birth and underwent a left-sided adrenalectomy, followed by lifetime substitution therapy with hydrocortisone.
May 2020. Admitted for a thymectomy.
May 2021. Admitted for a scheduled video-assisted thoracoscopic surgery to remove the lesions in the upper left lobe of the lung.
December 2021. Admitted for a radical right-sided subcutaneous mastectomy with a primary reconstruction of the breast with an endoprosthesis because of palpable lesions in the right breast.
February 2022. Admitted for a right lower lobectomy and atypical resection of the middle lobe of the lung due to adrenocortical carcinoma metastasis.

## Data Availability

Data used in this research are included within the article.

## References

[1] Zhao J., Tan Y., Wu Y. (2015). A rare case of eight multiple primary malignant neoplasms in a female patient: a case report and review of the literature. *Oncology Letters*.

[2] Zhao Z., Sun K., Yan T., Wei R., Guo W. (2020). Multiple primary tumors: a case report and review of the literature. *BMC Musculoskeletal Disorders*.

[3] Williamson C. W., Paravati A., Ghassemi M. (2015). Five simultaneous primary tumors in a single patient: a case report and review of the literature. *Case Reports in Oncology*.

[4] de Andrade K. C., Frone M. N., Wegman-Ostrosky T. (2019). Variable population prevalence estimates of germlineTP53variants: A gnomAD-based analysis. *Human Mutation*.

[5] Nandikolla A. G., Venugopal S., Anampa J. (2017). Breast cancer in patients with Li-Fraumeni syndrome - a case-series study and review of literature. *Breast Cancer*.

[6] Basile V., Puglisi S., Altieri B. (2021). What is the optimal duration of adjuvant mitotane therapy in adrenocortical carcinoma? An unanswered question. *Journal of Personalized Medicine*.

[7] Hisada M., Garber J. E., Li F. P., Fung C. Y., Fraumeni J. F. (1998). Multiple primary cancers in families with Li-Fraumeni syndrome. *Journal of the National Cancer Institute*.

[8] Macdonald O. K., Lee C. M., Tward J. D., Chappel C. D., Gaffney D. K. (2006). Malignant phyllodes tumor of the female breast: association of primary therapy with cause-specific survival from the Surveillance, Epidemiology, and End Results (SEER) program. *Cancer*.

[9] Birch J. M., Alston R. D., McNally R. J. Q. (2001). Relative frequency and morphology of cancers in carriers of germline TP53 mutations. *Oncogene*.

[10] Wu C. C., Shete S., Amos C. I., Strong L. C. (2006). Joint effects of germ-line p53 mutation and sex on cancer risk in Li-Fraumeni syndrome. *Cancer Research*.

[11] Hwang S. J., Lozano G., Amos C. I., Strong L. C. (2003). Germline p53 mutations in a cohort with childhood sarcoma: sex differences in cancer risk. *American Journal of Human Genetics*.

[12] Kamihara J., Rana H. Q., Garber J. E. (2014). Germline TP53 mutations and the changing landscape of Li-Fraumeni syndrome. *Human Mutation*.

[13] Kerlikowske K. (2010). Epidemiology of ductal carcinoma in situ. *Journal of the National Cancer Institute. Monographs*.

[14] Libé R. (2015). Adrenocortical carcinoma (ACC): diagnosis, prognosis, and treatment. *Frontiers in Cell and Development Biology*.

[15] Olivier M., Goldgar D. E., Sodha N. (2003). Li-Fraumeni and related syndromes: correlation between tumor type, family structure, and TP53 genotype. *Cancer Research*.

[16] Icard P., Goudet P., Charpenay C. (2001). Adrenocortical carcinomas: surgical trends and results of a 253-patient series from the French Association of Endocrine Surgeons study group. *World Journal of Surgery*.

[17] Luton J. P., Cerdas S., Billaud L. (1990). Clinical features of adrenocortical carcinoma, prognostic factors, and the effect of mitotane therapy. *The New England Journal of Medicine*.

[18] Sirianni R., Zolea F., Chimento A. (2012). Targeting estrogen receptor-*α* reduces adrenocortical cancer (ACC) cell growth in vitro and in vivo: potential therapeutic role of selective estrogen receptor modulators (SERMs) for ACC treatment. *The Journal of Clinical Endocrinology and Metabolism*.

[19] Fassnacht M., Johanssen S., Quinkler M. (2009). Limited prognostic value of the 2004 International Union Against Cancer staging classification for adrenocortical carcinoma: proposal for a revised TNM classification. *Cancer*.

[20] Kerkhofs T. M., Verhoeven R. H. A., van der Zwan J. M. (2013). Adrenocortical carcinoma: a population-based study on incidence and survival in the Netherlands since 1993. *European Journal of Cancer*.

[21] Chen H., Sippel R. S., O'Dorisio M. S. (2010). The North American Neuroendocrine Tumor Society consensus guideline for the diagnosis and management of neuroendocrine tumors: pheochromocytoma, paraganglioma, and medullary thyroid cancer. *Pancreas*.

[22] Kiernan C. M., Solórzano C. C. (2016). Pheochromocytoma and paraganglioma: diagnosis, genetics, and treatment. *Surgical Oncology Clinics of North America*.

[23] Bertherat J., Gimenez-Roqueplo A. P. (2005). New insights in the genetics of adrenocortical tumors, pheochromocytomas and paragangliomas. *Hormone and Metabolic Research*.

[24] Testa J. R., Malkin D., Schiffman J. D. (2013). Connecting molecular pathways to hereditary cancer risk syndromes. *American Society of Clinical Oncology Educational Book*.

[25] Engels E. A. (2010). Epidemiology of thymoma and associated malignancies. *Journal of Thoracic Oncology*.

[26] Mai P. L., Best A. F., Peters J. A. (2016). Risks of first and subsequent cancers among TP53 mutation carriers in the National Cancer Institute Li-Fraumeni syndrome cohort. *Cancer*.

[27] Scorsetti M., Leo F., Trama A. (2016). Thymoma and thymic carcinomas. *Critical Reviews in Oncology/Hematology*.

[28] Aprile V., Bacchin D., Korasidis S. (2021). Hypertermic intrathoracic chemotherapy (HITHOC) for thymoma: a narrative review on indications and results. *Annals of Translational Medicine*.

